# From Sociocultural Disintegration to Community Connectedness Dimensions of Local Community Concepts and Their Effects on Psychological Health of Its Residents

**DOI:** 10.1155/2013/872146

**Published:** 2013-06-04

**Authors:** Tom Sørensen, Robert Kleiner, Paul Ngo, Andreas Sørensen, Nils Bøe

**Affiliations:** ^1^Division of Mental Health and Addiction, Institute of Clinical Medicine, University of Oslo, Norway; ^2^Nordliveien 6, 1482 Nittedal, Norway; ^3^Emeritus, Department of Sociology, Temple University, Philadelphia, PA, USA; ^4^Psychology, St. Norbert College, De Pere, WI, USA; ^5^Research Assistant, Division of Research, North Coast Psychiatry, Nittedal, Norway

## Abstract

In a series of community mental health promotion studies in Lofoten, Norway, the concept of sociocultural integration is used to describe properties of a local community that are related to people's psychological health. Starting with Durkheim's description of a cohesive society, we compare different concepts that are related to sociocultural integration, for example, sense of community, social capital, and social cohesion. We then examine the relationship of various individual oriented social psychological concepts to sociocultural integration. These concepts often share theoretical and operational definitions. The concept of sociocultural integration in the Lofoten studies was proved to be very valuable in understanding how the properties of a community can affect people's mental health and their social psychological properties. It has also shown its value in the planning of mental health services and demonstrating its success in concrete community-based mental health promotion projects. Thus they could make important contributions to further studies and actions in local communities where the intersection between the individual, their social network, and their local community occurs.

## 1. Introduction

The seminal Stirling County study [1] took social psychiatry to a position where mental health promotion could be translated into concrete community involvement. From a social psychiatry that linked social stratification and social class to prevalence of psychiatric disorders [2, 3], Leighton amplified the ecological approach to include concrete community processes and to operational measures of the phenomena, thereby linking the context of geographic areas to specific characteristics, that is, the sociocultural integration of a local community to the occurrence and distribution of mental disorders. A main finding from the Stirling County studies was the stronger effects on mental health of higher levels of sociocultural integration of a local community than higher social class within those same communities [4, 5]. Taking off from Leighton's Stirling County study, Dalgard et al. [6, 7] and Sørensen [8] combined a psychiatric service perspective with an analytical focus on the local community that itself contributed to the mental health of a population. 

The major thrust of our basic and applied research project has been to contribute effective and sustainable strategies for treatment, intervention, prevention, and promotion of mental health and well-being, especially at the local community level. Unfortunately, the definition of “community” can vary from one social science discipline to another and even vary in the same discipline, making it difficult to evaluate and generalize from research using the concept. We defined communities in terms of geographical boundaries, that is, local communities, ethnic composition, or enclaves of people that may or may not have systematic interpersonal relations and obligations to each other. The main aim is to relate the concept of sociocultural integration-disintegration, as it evolved in the Lofoten mental health promotion studies to other concepts used to describe the local community and its linkage to mental health.

## 2. Lofoten and the Lofoten Mental Health Promotion Studies

The Lofoten region in North Norway has about 24,000 inhabitants and consists of a series of islands stretching out into the Gulf Stream north of the Arctic Circle. The region is composed of six municipalities. The main income of this region has historically come from the Norwegian Arctic cod fisheries in the wintertime. Work connected with the fisheries has been important in all of the local communities we have studied. In the six Lofoten municipalities, peopled employed in the fisheries varied from 7,7% to 30,7% when the Lofoten mental health promotion studies were started in the early 1990. But in the two largest municipalities, more people were employed in both industry and health and social services. In the last year of the 1980s, the usual migration of cod that come to spawn along the coasts of Lofoten in February-March failed to occur. The authorities introduced limited quotas that could be taken by each boat. That put the fishermen, boat owners, and the fishery companies ashore into an economically impoverished situation. The mental health promotion project was derived from this fishery crisis.

The present studies from Lofoten in North Norway are in debt to Leighton's comprehensive approach to the mental health of communities, (a) mapping the need for psychiatric attention/services, (b) analytically relating them to community functioning, (c) developing and describing mental health services in a small rural community [9], and (d) carrying out mental health promotion projects through community development [10]. The Lofoten projects were, in the main, evaluated by surveys designed to interview total adult populations. Indicators have been developed that show the effects of project priorities, the program targets, and measures of progress, that is, having mental health promotion indicators at individual, organizational, and community levels. The ultimate objective of such a focus is to identify the social and psychological capital (or resources) in each community that can be mobilized to deal with the psychological problems that exist in their respective communities, that is, factors of importance for promoting mental health and preventing mental illness.

A group of mental health promotion projects named “Liv Laga in Lofoten” were carried out during the 1990s using as points of departure the Lofoten psychiatric outpatient clinic. Early in the project, “Local Community Development” focused on the entire adult population of seven selected local communities. The Lofoten studies were at the intersection of a theoretical approach to the community-individual mental health interaction and the practice of evaluating and setting into action mental health promotion in local community contexts. The impact of local communities and the relevance of sociocultural integration to living conditions and mental health can best be seen in communities like Lofoten where the present studies were carried out. Here, people have most of their culture, social life, work, and, for some, even family within the same geographic area.

For bad and good, inhabitants of small communities as in Lofoten are more affected by the level of sociocultural integration. In more urbanized areas people would usually have their work and recreational activities outside their residential neighborhood, and their personal network would often have many locations. If a crisis occurs, it would not necessary hit all important supportive structures at the same time. On the other hand, efforts to change a neighborhood would not have the same total impact as could be done in smaller local communities like Lofoten. Hence, the generalizations from the Lofoten studies are most relevant for smaller local communities with a relative high overlapping of social network sectors.

## 3. Sociocultural Integration-Disintegration and Its Application in Lofoten

Leighton, in “My name is Legion” [1], the first book on the stirling County Study of psychiatric disorders and sociocultural environment, gives a detailed theoretical framework for the connection between the sociocultural structure of a local community and the amount of psychiatric disorders among its inhabitants. He looked at a community as a self-integrating and self-regulating unit or “quasiorganism.” This “functional” point of view reflected an orientation deriving in part from anthropology, for example, Radcliffe-Brown [11] and Malinowski [12], and also from sociology, for example, Merton's [13] and Hyman and Singer [14] discussions of anomie and reference groups, and Parson's [15] emphasis on the “systems approach.” Other writers discussed this strategy in terms of consensus (trend towards agreement on orientations or meanings) and functional interdependence (complementary interactions and exchange of goods and services) in social systems [16, 17] or used concepts like organization-disorganization [18]. 

In the work done by Leighton, integration-disintegration is treated as one dimension, and communities (in Nova Scotia) could be ordered according to their sociocultural integration-disintegration. He listed types of factors that affect the integration-disintegration. Initially, “causes” for disintegrating the community were (a) a recent history of disaster, (b) widespread ill health, (c) extensive poverty, (d) cultural confusion, (e) widespread secularization, (f) extensive in and out migration, and (g) rapid and widespread social change. Later, a second set of “effects” of disintegration included (i) high frequency of broken homes, (ii) few and weak associations, (iii) few and/or weak leaders, (iv) few recreation activities, (v) high frequency of hostility, (vi) high frequency of crime and delinquency, and (vii) weak and fragmented networks of communication. Looking at these “causes” and “effects” showed to us that integration-disintegration is multidimensional and not unidimensional [19].

Based on surveys of seven local communities in Lofoten, factor analyses of statements evaluating the local community identified eight factors or dimensions to the integration-disintegration concept. Four dimensions were related to the core community integration functions: initiative and cooperation, leadership, community identity, and contact and communication, and four were related to risks and resources in the community: future economic viability, leisure time and recreation, milieu for children and youths, and security when sick or old. Each statement included the name of the respondent's actual local community. 

From our own experience, the core of the disintegration process is the disruption of social communication systems and structures of mutual assistance. The signs are the absence of functional leaders and people with ideas and initiatives, with a lack of people to follow such ideas when they are put forward. The community viability is reduced with economic problems, dissolving families and other formal and informal organizations, deteriorating interpersonal relations, and the weakening of norms about what is right or wrong undermining their physical, psychological, and social security. These changes in a local community do not make a place for growth, self-realization, acknowledgment, recreation, and being together with their significant social network(s). The total picture is one of increasingly burdensome situations with increased risk of mental problems on the individual level.

The “Local Community Development Project” in Lofoten focused on the entire population of seven selected local communities. The activities that evolved illustrate what emerged in the context of the seven Lofoten municipalities at a time of economic difficulties in the fisheries. The selection of sites for local community development had, as its point of departure, the ongoing fishery crisis. Hence, fishery-related local communities were picked. The explicit criterion brought forward in the dialog between the municipality and the project leader was to have the local communities represent all dimensions and structures of the fisheries, including its links with farming. Each of the selected communities had a history, an identity, a name, geographical boundaries, and an “informal” social structure. They actually constituted “the old school districts” that have (or have had) an elementary school within the borders of the community (the old school house could now be used as a community center). Often the community also had a church building and a graveyard.

The context and the planning process was an essential part of the health promotion strategy. The Lofoten project was given legitimacy from two sources, (a) from key authorities in the region, by having a series of meetings with people involved in health and social services, and (b) by meeting with politicians and administrators in each of the municipalities in the Lofoten region. Ideas for health promotion measures were discussed and translated into concrete plans. A second step was to have the overall project has its office based within the regional political liaison body, that is, “Lofotrådet." The focus on the interrelation between mental health and the totality of people's life context was also reflected in the composition of the steering group of “Liv Laga i Lofoten,” which included having the mayor in one of the municipalities as its leader. Several different initiatives occurred in the same population in the same time period, stimulating interactive processes. The activities were not to be fully defined at the start of the project. They had to allow for concrete input from the various segments of the community as an inherent part of local mobilization. In this way, one could secure the target groups' or locals' feelings of ownership, identification with the project, and be responsive to real local needs, and thereby also promote leadership and cooperative competence.

The “Local Community Development Project” was clearly focused on such locals' feelings of ownership and identification. After meeting the municipal leaders, the first initiative in the community development process was to contact key community members (in each of the chosen local communities) by visiting the community, getting to know people, and gathering information about their local community. Thereafter an informal local working party was established with members of the key local social network(s). The first task of this group was to systematize the information that would be gathered, especially with regard to what was of value in the local community, what should be preserved, and what was wanted. The next initiative was to conduct open “popular meetings,” to invite all local inhabitants, and particularly to encourage attendance by people who would be stimulated in the project to actively participate in working groups. These “popular” meetings were to be “idea workshops” based on various themes, including culturally defined issues, family issues, local environmental problems, tourism, trade, and industry. The participants concluded the meetings by assigning priorities of the ideas brought forward. The third step was based on the work done in the “popular” meetings and focused on establishing a formal organization whose aims would be derived from the priorities, or themes, decided upon at the meetings. Working groups around thematic projects were established. Reports from various initiatives were brought back to subsequent meetings.

In each of the seven Lofoten local communities, surveys were carried out to acquire information from residents to be used in the community development programs, as well as data to evaluate possible changes in both personal mental health and in properties of the community structures, and hence three cross-sectional surveys of the same seven local communities were carried out. The followups were linked by community and not by individual. The T1 surveys of the local communities that participated in the mental health promotion project took place from June 1991 to May 1992, namely, within the first few months of the community activities. The followup of all seven communities, that is, at T2, was carried out in 2000 and a third T3 survey took place in 2010. No externally organized community interventions took place after 1994. To varying degrees, project activities were continued on a local basis. 

## 4. Comparing Concepts—The “Cohesive Community” in Perspective

Currently relatively few researchers explicitly use Leighton's integration-disintegration concept, but the literature is filled with references that make use of different concepts or terms that sound different but, in reality, have similar meanings to our perspective. Conversely, there are also many references that make use of the same concept or term but the actual meaning of the term differs from one group of researchers to another. This situation makes it difficult to integrate such material except at some vague theoretical level. Too often we find ourselves superimposing a vague general unproductive perspective on the literature. We need clearly defined concepts that have importance for our perspective even though they may be seen and used differently in the literature.

Historically, the perspective of societal disorganization causing personal disorganization was put forth by the French philosopher Comte and applied specifically to psychiatry by Audifferend. The opposite position, namely, that personal disorganization caused disturbances of the society, was put forward by Bastide [20]. A starting point for defining a community could be Durkheim's [21, 22] description of a cohesive society, that is, a society characterized by a multitude of mutual moral supports, which does not depend on individuals own resources but leads them to share their the collective energy and mutual support. Durkheim defined the cohesive society in terms of mutually defined and agreed upon role expectation and goal directed behavior which would lead to positive mental health. Tonnie's division of social groups into “gemeinschaft” and “gesellschaft” [23] is also a forerunner of the concept of the “cohesive community.”

The Chicago School of Sociology [24] conducted early significant empirical investigations using the community ecological approach focusing on the effects of selected social properties of environment. Faris and Dunham [25] mapped the distribution of mental disorders by various characteristics of communities in the urban environment. Influenced by the Field theory perspective, Parker and Kleiner [26] have demonstrated the potent effects of considering simultaneously the interaction of psychological, social, and objective properties of the situational context on mental health status and mental disorder. Weinman and Kleiner [27] have shown how the deliberate manipulation of the community context in which groups of patients (i.e., patient networks) live influences therapeutic success and the mental health status of the patients. The variables they explicitly dealt with included group cohesion, social support systems, and methods of conflict resolution.

Leighton emphasized that increased economic and educational opportunities were not enough to bring about a turn for the better in a disintegrated community. One needed the development and enhancement of socially important processes: leadership, followership, and practice in cooperatively working together, thus enabling people to gain confidence that they could do things to better their lot. This could perhaps explain the findings from research on regenerated neighborhoods in the UK [28], which illustrate how difficult it is to shift the perception of a community's residents and the intermediary agencies, and the finding of no mental health improvement following the urban regeneration project [29]; that is, bettering in the standard of living is not enough.

Social disorganization could be defined as the “inability of a community structure to realize the common values of its residents and maintain effective social controls” [30]. This approach also sees local communities and neighborhoods as complex systems of friendship, kinship networks, and formal and informal associations, rooted in ongoing family life and socialization processes [31, 32]. Clearly they have a lot in common with Leighton's view and the views of other researchers that have focused on the cohesive community.

Gusfield [33] said that “territorial” dimensions of communities are an oversimplification. One needs the relational dimensions as well. Some communities, like local communities in this paper, are defined primarily according to territory, but even proximity or shared territory may not by itself constitute a community. Factor analyses of an urban neighborhood yield two distinct factors [34], named social bounding and physical rootedness, similar to Gusfield's dimensions above. “Together with the family, the neighborhood is one of the few places where a community can emerge without external intervention [35]”.

For us, social capital [36] emerges as a meaningful concept because it embraces individual (internal) and social (external) resources, the degree of sociocultural integration, and the interaction of both types of resources. Social capital is often used in a narrower and simpler way than the way we intend to use it. Although in the literature, social capital may be defined in terms of any of the following properties: (a) values of social networks, (b) the bonding of similar people, (c) linking diverse people with norms of reciprocity [37], or (d) properties that facilitate collective action [38–40]. But definitions can also consider internal cognitive factors aspects of social capital which also describe community characteristics, that is, degree of trust, reciprocity of norms, and the quality of interpersonal bonds [41].

In our view, a geographically defined community is socially integrated or cohesive to the degree that it has internal and external social capital. A cohesive community can be described as a society with a high shared sense of morality and common purpose, aspects of social control and social order, high level of social interactions within communities, and a high sense of belonging to a place [42]. Coleman [43] introduces the concept of social capital and underscores its effects on the formation of human capital in a community, and how social capital, especially social cohesion, is linked to the health of communities. Other studies have also dealt with social capital and health [44–46].

In sum, researchers mainly describe five domains of social cohesion in geographically delimited rural communities and urban neighborhoods. They include (a) common values and civic culture, (b) social order and social control, (c) social solidarity and reductions in wealth disparities, (d) social networks and social capital, and (e) place attachment and identity. Hence, social cohesion, broadly considered, would encompass most of Lofoten study's four core dimensions related to community integration, as well as their relation to mental health.

## 5. Sense of Community and Other Place Related Concepts

A concept clearly related to social integration is the “sense of community” [47–49]. It has been used in local community studies and in projects dealing with “planned healthy communities” [50]. Farrell et al. suggested that this construct includes the perception of similarity to others, an acknowledged interdependence by giving to or doing for others, expecting the same from others, the feeling that one is part of a larger dependable and stable structure, that is, the capacity of community members to influence each other, and the interdependence of the individual and community. More developed local communities encourage the development of a sense of community among their community residents. The absence of a sense of community has been found to bring about feelings of alienation, isolation, and loneliness [51], while a strong sense of community has been linked to a range of positive outcomes including improved well-being, empowerment, sense of efficacy, life satisfaction, and happiness [52–54, 54, 55].

Other definitions are given in the literature that vary in complexity of operational definition but are analogous and even partially the same. For example, McMillan and Chavis [56] included four dimensions: (a) membership under which resubsumed boundaries, emotional safety, a sense of belonging, and identification), (b) ability to influence others or being influenced by the others (c) integration and fulfillment of needs bytheir participation in the community, and (d) shared emotional and participating linkages to a shared history. Obst et al. [57] added “conscious identification” as a fifth dimension. Unger and Wandersman [48] suggested that sense of community includes three components: a social component (emotional and instrumental support and social networks), a cognitive component (cognitive mapping of the physical environment and symbolic communication), and an affective component (the emotional attachment individuals have to persons living around them). This specification seems clearly related to Leighton's concept of integration-disintegration.

Place attachment can be described in the same terms as the bonding that occurs between individuals and their important environments [58, 59]. Some authors use sense of place as a somewhat broader concept, that is, sense of community as one dimension [60] and place attachment as the other. Here sense of community is defined as the residents' place attachment. Overlapping place related descriptions would be place dependence and place identity. Place attachment has been used in studies of mental health behavior among adolescents [61]. Other related concepts are “sustainable communities” [62] and “community connectedness” [63, 64].

## 6. Individual Capital and Social Capital

Social capital is clearly a community level concept. The way the Lofoten studies have measured it also sees it as a community level concept. All descriptive statements refer to the actual local community (using its name), and the data is analyzed in terms of aggregate properties. Human capital, that is, individual psychological resources, is analyzed together with social capital. In the Lofoten study, social support [65], sense of coherence [66, 67], empowerment [68], self-esteem [68], self-efficacy [69], and mastery [70] would be examples of such human capital related to psychosocial coping behavior. Social capital is defined in terms sociocultural integration of a local community. Social capital could influence and interact with human capital expressed as sociopsychological resources. In mental health promotion projects, such individual factors could be important intervening variables that enhance the development of the community's capacity to deal with such problems.

### 6.1. Social Support

The perception of “social support” in one's informal social networks and its relation to the integration of the network is important. Originally the construct was seen only from the individual perspective and not reflecting on the local community or social networks social support as a system in its own right. For example, Keyes and Shapiro [71] define social well-being as an individual's self-report of the quality of his or her relationship with other people, the neighborhood, and the community. Lindenberg [72] also speaks of community when individuals realize multiple well-being goals within their group. His description of social well-being, as feeling recognized and accepted by others and being liked, and receiving confirmation for one's behavior is similar to the definition of social support we have used in the Lofoten study [73]. The distribution of personal expectations of social support could also be used as an indicator of social capital in a community when individual data is collectively analyzed and seen as a reflection of property of the community [74]. Social support enables individuals to use more effective coping strategies that facilitate problem comprehension and action, thereby reducing emotional distress [75]. At the community level this, could measure potential resources for meeting life events that affect a significant part of its population, and/or the community as a whole.

With a community focus, social support, in terms of concrete collective assistance, also acts to cope with the emergence of risks, stresses, the escalation of problems, and structures that influence such processes. With adequate resources within a local community, a possible risk situation may be resolved and not be experienced as a stressful life situation. If this network action is recognized by the individual, it would manifest itself as a positive main effect of social support in relation to mental health [76]. In the surveys in Lofoten, we noted substantial shared contributions of personal social support and the dimensions of sociocultural integration in relation to mental health and well-being [73]. Two items in the social support index, that is, “belonging to a group,” and “help when sick”, were most potent among the local community properties.

### 6.2. Sense of Coherence

Antonovsky introduced “sense of coherence” and defined it as “the extent to which one has a pervasive, enduring though dynamic feeling of confidence …” [77], that is, a personality-related factor. It is clear from this definition that it is the person's relationship with both his/her internal and external environments that determines the degree of this factor. He also proposed that this feeling of control is shaped by the influence of collective evaluation of one's important social networks [78]. Other authors proposed the reverse direction of causality, that is, people with a strong sense of personal control use their social network(s) more often and more efficiently, thereby affecting their perceived social support [79, 80]. A stable community, providing stable social support, could directly enhance the development of a stronger sense of coherence among its inhabitants [81]. In sum, the sense of coherence, even if primarily a personality-related resource can be influenced by the integration properties of the community, but even more important is that an individual's psychological makeup can contribute to community development. The latter can have favorable potential for key networks within a local community. Subsequent analysis from Lofoten found social support and sense of coherence to have independent, overlapping, and interactive components with regard to mental health [76].

### 6.3. Empowerment

Rappaport linked the concept of “empowerment” to community psychology. It leads to collective actions to improve the quality of life in communities and to important linkages among community organizations [82, 83]. For Rappaport the aim of empowerment was to enhance the possibility for people to control their own lives. Thus empowerment was defined as a process: the mechanism by which people, organizations, and communities gain mastery over their own lives [84]. Psychological empowerment may generally be described as the connection between a sense of personal competence, a desire for, and a willingness to take action in the public domain [85, 86] or as a social-action process that promotes the participation of people, organizations, and communities towards the goals of increased individual and community control, political efficacy, improved quality of life, and social justice [87].

Rogers et al., using a factor analytic procedure with their “Making Decisions Empowerment Scale,” identified five dimensions of empowerment, that is, self-efficacy/self-esteem, power/powerlessness, community activism, righteous anger, and optimism/control over the future as key components [88, 89]. This was a point of departure for the empowerment concept in the Lofoten study. In our analyses of the Lofoten surveys, we identified four factors, resembling, but not identical with Rogers' factors. These were community activism, power/powerlessness, self-determination, and self-esteem [90].

More recent definitions have made important distinctions between the subjective experience of psychological empowerment and the objective reality of empowered community structures. Psychological empowerment can be defined as a feeling of greater control over one's life and may occur without participation in collective political action. Community empowerment includes a heightened level of psychological empowerment amongst its members influenced by collective political action, or the achievement of favorable redistributions of decision-making resources in the community.

## 7. Measuring “Community”

Communities can be studied by focusing on people's emotional sentiments towards their community and/or by using a more rational community evaluation [91, 92]. Leighton's anthropological approach of measuring communities' degree of integrated focused on (a) emotional sentiments towards the community, that is, social cohesion, and (b) more rational evaluations of risk and resource factors. A similar approach was used in the Lofoten studies. Many approaches to the community look at neighborhoods as communities measured by the number and quality of relations to neighbors [93, 94]. The most systematic behavioral approach has been the study of behavioral settings, places where people in a community meet and manifest social interaction as suggested by Barker's work in ecological psychology [95, 96]. Measurement of “sense of community” focused on how people experienced their community [97]. They did this by asking questions about individuals' perception, understanding, attitudes, feelings, and so forth about community, and how they relate to it. This is for example, measured by the Neighborhood Cohesion Index (NCI) [98, 99]. But this scale does not so much enquire for risks and resources.

The various approaches in the field tend to depend on methods that are specific to a discipline and where the data may be empirically narrow or superficial. Earlier, we made the point that Leighton's studies depended on anthropological methods, but it is also important to indicate that other methods were necessary to make sure that this position was justified. For example, they used individual interview surveys based on sampling procedures, and medical records to investigate people's mental health. This was clearly necessary, as well, in the Lofoten studies. However, the main method in Lofoten depended on survey data that gathered the total adult population in each community. We analyzed the changes in the data from three points in time in each community. However, we found that other sources of data were necessary to evaluate the effectiveness of our projects. More specifically, popular meetings in each community occurred at the beginning of the period and during the project period. Detailed notes were kept and audio tapes were made during the entire process and at all community and committee meetings. Newsletters were published in the same period. After the project terminated, there were also systematic interviews at meetings with key persons in each community to give feedback about their experiences during the follow-up periods. The project coordinator for the total project was interviewed systematically about the project and other developments in each of the communities.

## 8. Community Promotion and Mental Health

Targeting the local community in mental health promotion was the optimal place for dealing with intimate personal concerns and the wider sociopolitical context [100]. In the Norwegian context, psychiatric health services have treatment responsibilities related to fairly small geographic areas and hence focusing on community mental health promotion was feasible and could be part of the practice. A study of how a particular local community, “The Road” in Nova Scotia, manifested a decrease in mental problems when it was deliberately transformed from a disintegrated to an integrated community [10] argues for the validity of a causal relationship between sociocultural integration of a local community and mental disorder. Leighton emphasized that increased economic and educational opportunities are not enough to bring about a turn for the better in a disintegrated community. One also needs the development and enhancement of socially important processes, that is, leadership, followership, and practice in working together cooperatively. This would enable people to gain confidence that they can do things to better their lot.

In Lofoten, following community integration efforts, we also had a community showing a parallel process towards higher sociocultural integration and a concomitant augmentation in psychological health [101]. In addition to the six communities that participated in the local community development projects at T1, one local community approached the project leaders on its own. They asked for help to construct a community profile of its problems and assets. They felt their local community was in danger, because they could lose their school and other institutions that they felt was part of a living community. During recent years, the population had declined and many services had moved to the more central parts of the municipality. In sum (at T1), we could observe a process of societal disorganization. In the intervening time, T1-T2, this local community was exposed to two opposing fields of forces. Resources like school, shops, and other services were closed down or moved to more central parts of the municipality. On the other hand, through actions to save and further develop a home for the elderly, many of the inhabitants came together and through cooperation succeeded in their “struggle.” People from this community participated in courses about care for the elderly, including training to create organisations and for developing leadership skills. The home for the elderly also developed into a community centre, bringing together people for voluntary communal work, including an organisation to attend to the needs of the residents of the institution, as well as provide recreational activities for the general population. The development was compared to six other local communities in Lofoten. Of the seven investigated local communities in the first series of surveys (T1), this community had the highest mean (or score) on nervous symptoms, the second lowest on well-being, and the lowest perceived social/support. At T2, all these three indicators positively increased and got close to the mean Lofoten scores. Compared to the other investigated communities in Lofoten at T1, the inhabitants also placed themselves low on most of the indexes of community integration. This indicated a low level of sociocultural integration. From T1 to T2, we observed an increase on the majority of the indexes. Although the increase was often not significant when we looked at the indexes one by one, but the tendency seemed clear. At T2 this community had moved closer to the level of the other local communities in Lofoten, hence, a development towards higher integration.

Looking at two other communities in the project which were highly integrated at T1, that is, in the early phase of the intervention project, one remained integrated, even increasing, over the nearly 20-year follow-up period, and there are indications that the mental health promotion project contributed to this development [102]. The process of how the objective reality targeted the social, and subjective reality could clearly be seen in the second of these communities. The decline of this community seemed to follow the construction of a bridge that connected the community with the municipal center, that is, how a particular geographical change could disturb the economic base and social organization of this local community. The first community could be seen as reflecting the opposite causal process. The cohesive social relations helped a community make the most of its geographic context and developed the community with positive changes in all three realities. A main asset in this first community was a very collective leadership group. Many of the original project leader group meets with us at follow up meeting years later. But in the process after the formal end of the project, other people were also taking part as leaders in different local organizations. An important aspect of the social reality was that this second community was not dependent on one person to make the project work; that is, there was reduced dependency on any single person. In the second community, leadership leaned heavily on one person and some few in her network. When this group had personal difficulties or moved, other did not take over and brought the activities further. In sum, it points to the need to know the characteristics of the community at the start of the project and emphasizes the need to involve the community as a whole in the project as quickly as possible in the planning and decision making.

Authors who have used the disintegration concept in mental health promotion have had different targets to change [103]. To some, it has been the (a) entire population, especially in terms of developing leadership and cooperation as in the Leighton and present project [104], (b) community clubs aimed at community image and community decision making [105], or (c) engineering projects in isolated communities with a simultaneous focus on community participation and social integration [106]. Dalgard, in a series of studies [107, 108], has developed community health profiles that were either presented to authorities of the given communities or directly used in projects with risk groups that manifested increased symptom levels [109, 110].

In a study by Latkin and Curry [111], perceptions of one's local community, as indicators of social disorganization and social stress, predicted depressive symptoms at a 9-month follow-up interview, suggesting the need for structural intervention in local communities. On the individual level, social integration was related to plasma concentration of fibrinogen among elderly men, which may help explain the association between social integration and mortality in men [112]. A study that examined the interaction of structural characteristics of a neighborhood and the subjective experience of social cohesion revealed that high levels of community social cohesion reduce the adverse effects of small-area deprivation on mental health [113–115]. The perception of higher levels of cognitive social capital (e.g., trust in neighbors) is associated with a lower risk of developing major depression during a 2-3 year followup [116].

A report from the University of Glasgow [42, 117] describes eight domains of social capital. In relation to these domains the researchers list appropriate policies to support them. Many of these would be consistent with the experience in Nova Scotia and with our own projects in Lofoten, providing support to community groups, helping to provide solutions to problems, giving local people a voice and a role in policy processes, establishing and/or supporting local activities and local organization, developing and supporting networks between organizations in the area, securing harmonious social relations, promoting community interests, encouraging trust in residents in their relationships with each other, bringing conflicting groups together, encouraging a sense of safety in residents, and boosting the identity of a place. In itself, insufficient sense of community is seen as a barrier to community development [52, 97, 118–120]. In a study from Northern Ireland [121], the successful intervention in rural communities, much like the experience in Lofoten, was characterized by “a partnership model of working; local coordinating structures and consultation mechanisms; use of structured planning model to guide program planning and implementation; mobilization of cross-community and inter-agency support; and a comprehensive logic evaluation framework to assess the input, process, impact and outcomes of the project as it unfold”. 

A review of the literature [35] identified at least four major dimensions that stimulate the creation of community: (a) meeting opportunities, (b) individual motivation to invest in others in the group, (c) alternatives to realize individual goals, and (d) interdependencies. Important for the creation of community was interdependence among neighbours, the intention to stay in the neighbourhood (e.g., people with young children), more facilities and facilities related to the market (e.g., shops), homogeneity with respect to income, and ownership of one's home. On the other hand, having more relational alternatives outside of the neighbourhood can work against the creation of community.

In the Lofoten studies, Lewin's concepts of life space and social space [122], even if not explicitly formulated at the planning stage, have been important for our understanding and study of the “psychology of local communities.” “Liv Laga i Lofoten” has been an action research project, having seeker conferences, quality circles, and the building of cooperative networks in its toolbox. Hence, the practical implementation of the projects in Lofoten is not only in debt to Leighton, but also to Lewin and his students. Lewin and his students laid the early foundation for understanding change in social situations [123–125]. Lewin's model of planned change [126–128]; has added a useful analytic tool for the further development of the Lofoten mental health promotion approach. Furthermore, Lewin believed that organizations, with their associated patterns of attitudes, expectations, and behavioral forms, behave much like other living biological systems and are influenced by the concept of homeostasis. This has the same connotations as Leighton's descriptions of the integrated/disintegrated community as a quasiorganism. Lewin's planned change could be at template for local community mental health promotion. This approach brings together four integrated elements: *field theory* which seeks to map the totality of human behavior that is taking place; *group dynamics* which seeks to understand the behavior of groups; *action research* which requires analyzing the situation and choosing the best change for the situation; a *stepwise change model* (unfreezing present stagnant or negative community behavior; learning and effectuating new behavior; stabilizing the new equilibrium).

## 9. Some Concluding Reflections and Future Directions

What have we learned from the mental health promotion project in Lofoten? Is this strategy a feasible way to promote mental health? What types of local communities can make the most of this method? Would the impact of local communities on mental health vary along a “gemeinschaft-gesellschaft” dimension, that is, would community integration have a larger impact on people's well-being and psychiatric status if it is nearer to the “gemeinschaft” end of such a dimension? Our preliminary answers to these questions are in the affirmative. Our principles for increasing the integration of a local community deriving mainly from Leighton's studies were the guidelines for the health promotion activities and the nature of the research. The dynamic pattern of the “Liv Laga Model for Mental Health Promotion” is supported by an urban study [129], showing that individual factors like perceived stress, depressive moods, and situational determinants like undesirable life events were related to a decrease in social integration in the community. Interpersonal determinants such as positive emotional guidance and instrumental support were associated with increases in social integration in the community. Hence the Lofoten project of combining activities that reduce stressful life burdens with enhancement of social interaction should be a comprehensive way of doing mental health promotion on the community level. 

In the Stirling County studies, Leighton evaluated the effects of sociocultural integration on mental health. His theoretical approach was the basis of a mental health project that transformed a small community, “The Road,” from a poorly integrated community into a well-integrated one. The qualitative measurement methods used by these anthropologists working with Leighton may lack precision which may explain the hesitation to use them in other studies of mental health promotion projects. The strength of the data gathering procedures used in the Lofoten studies is the inclusion of subjective and collective evaluations about the community, that is, giving questionnaires constructed for self-completion to the total adult population. This gives collective rational community evaluations of the problems, risks, and resources. The use of self-completing questionnaires in a community is a feasible way of overcoming the problems of sampling methods. By delivering questionnaires to all adult inhabitants in a local community it was possible to get closer to the universe of respondents in each community. Repeated surveys over time in the Lofoten project provided feedback on the effects of concrete actions initiated in promotion projects. The quantification of the community indexes allowed us to measure the degree of change in community promotion projects. The dimensional properties of the questionnaire open up for more specific actions and evaluations.

In the studies in Lofoten, it became clear that evaluations of the community projects depended on several types of data and analyses. We had to analyze the change over time in the importance of each dimension of community integration, the changes in each of the outcome measures, and the changes in community consensus. It was also necessary to obtain notes and records from group meetings and evaluative comments from the formal and informal leaders in the community on the perceived effects of the strategy in their community. Success (or failure) of a community project could show itself in a myriad of different ways.

In 2010, following the Lofoten project, 12 local communities in Valdres in southern Norway were selected for study. Valdres consists of six municipalities in a rural mountainous region. In each of these municipalities, two communities were selected by local authorities. In each municipality those communities were chosen with regard to having contrasting resources and risk characteristics [130]. After the surveys, meetings with each of the six municipal councils were held. They focused on how the population scored on the measures of mental health and on the indexes of community integration which differed among the selected communities. In Valdres, they compared them to the communities in Lofoten. Particular attention was paid to the meaning of different patterns of means and correlations in the communities, combinations that suggested possible targets for community interventions. The discussion with the members of local councils supported the use of means and correlations as a way of defining health promotion activities. Comparing Lofoten to Valdres also allowed us to determine the effects of different geographical characteristics on social and psychological processes. The comparison between the Lofoten programs with the planning for Valdres communities opened up new opportunities for translating theory and methods into more sophisticated and successful uses of community resources.

In recent years, similar studies have begun to follow the strategy described here and have used some of our data gathering methods. A study of urban regeneration and mental health in Manchester [131, 132] used some of the most potent Lofoten items. They found that higher overall quality of life ratings were associated with greater sense of belonging to the community, less feelings of isolation, better leadership, and more leisure opportunities. Lower subjective quality of life was associated with the feeling that the area was in decline. Several questions measuring community integration and connectedness from the Lofoten studies were also used in a Norwegian study called HUNT2, conducted in the county of Nord-Trøndelag. Their index of community connectedness was also shown to be related to subjective well-being and self-rated health [133]. 

The Norwegian studies have shown that the concept and the measuring of sociocultural integration are very valuable in understanding how the properties of a community can affect people's mental health and their relation to other social psychological concepts. It has shown its value in the planning of mental health services and has demonstrated success in concrete mental health promotion projects. The concepts and the methods used in the Lofoten studies could make important contributions to further studies and actions in local communities where the crossroad between the individual, their social network, and their local community occurs, that is, an extension of Dalgard's concept “community mental health profile” [134].

A main strength of the sociocultural integration-disintegration concept is the possibility to integrate an emphasis on risk and resource factors of the community's objective reality with a more long lasting experience of a community that has or has not sufficient structural capacity to support and maintain people's need for social support. Leighton's theoretical proposition of looking at a community as an entity that is more than the sum of its individual members strengthen a mental health promotion strategy that encompasses more than detection of individuals at risk. The strength is also the very concrete link between theory and practical actions. This theory-action linkage is even more developed and directed at specific domains in the Lofoten project. 
